# Knowledge regarding Zika Virus Infection among Healthcare Providers in an Academic Tertiary Care Center in Riyadh, Saudi Arabia: A Cross-Sectional Survey Study

**DOI:** 10.1155/2020/8145219

**Published:** 2020-03-11

**Authors:** Mohammed Alessa, Mohammed Alzahrani, Abdulmajeed Alshehri, Amjad Aljrboa, Rami Bustami, Abdullah Almangour, Abdulaziz Alsalem, Jawaher Gramish, Moteb Khobrani, Thamer A. Almangour

**Affiliations:** ^1^Department of Clinical Pharmacy, College of Pharmacy, King Saud University, Riyadh, Saudi Arabia; ^2^College of Pharmacy, King Saud Bin Abdulaziz University for Health Sciences, P.O. Box 3660, Riyadh 11426, Saudi Arabia; ^3^King Abdullah International Medical Research Center (KAIMRC), Riyadh 14611, Saudi Arabia; ^4^Pharmaceutical Care Services, King Abdulaziz Medical City, Riyadh, MC 1445, Saudi Arabia; ^5^Department of Clinical Pharmacy, College of Pharmacy, King Khalid University, Abha, Saudi Arabia

## Abstract

**Background:**

Zika virus (ZIKV) has become a major concern across the world. It is highly necessary for healthcare providers (HCPs) to have sufficient knowledge about such a disease. The purpose of this study is to assess the knowledge regarding ZIKV among HCPs in Riyadh, Saudi Arabia. *Materials and Methods*. A cross-sectional survey study was conducted at a tertiary care center in Riyadh, KSA, during a two-month period from September 19, 2016 to November 19, 2016. Descriptive statistics were performed on data collected. For continuous variables, data were expressed as means ± standard deviations (SDs), medians, and ranges. Proportions were used to describe categorical variables. Knowledge scores were evaluated and compared by demographic characteristics including age, designation, years of practice, and gender, using the *t*-test/Mann–Whitney *U* test or the Kruskal–Wallis test, as appropriate.

**Results:**

A total of 336 HCPs from different specialties (physicians, dentists, nurses, pharmacists, and nutritionists) completed the questionnaire. Significant differences in knowledge about ZIKV were observed by participant's age. Significantly higher knowledge levels were observed among older participants (45 years or more; *p*=0.011). A substantial difference in the knowledge level was observed by department, with pairwise comparisons showing significant differences in knowledge scores between all departments except for Pharmacy vs. Nutrition and Nursing vs. Internal Medicine. Knowledge scores were not significantly different by years of practice.

**Conclusion:**

Our study showed inadequate knowledge of HCPs from different specialties about ZIKV infection which needs to be improved in terms of infection prevention and control. Awareness about ZIKV infection should be ensured and maintained among HCPs to face any possible emergence in the region.

## 1. Introduction

Recently, the Zika virus (ZIKV) has become a major concern across the world. It is a virus which belongs to Flaviviridae family [1, 2]. It was first isolated from a monkey in 1947 in the Zika forest of Uganda, and the first isolation of the virus from a human was also found to be in Uganda in the 1960s [3, 4]. Later, ZIKV was discovered in human populations in many countries including Sierra Leone, the Philippines, Indonesia, and India; however, it was not a major public concern at that time [3–5]. Attention to ZIKV reached a global level after an outbreak in Latin America in 2015 [2]. ZIKV can primarily spread through the bite of infected mosquitoes, especially the *Aedes aegypti* type [1, 2]. In addition, this virus can be transmitted through sexual contact or by an infected pregnant woman to her fetus, which may lead to microcephaly, a serious birth defect of the brain [3, 6–8].

Lately, cases involving ZIKV were reported in more than ten countries in the Americas region, which indicated the rapid spread of the virus [9]. An accelerated spread of the virus increases the demand of taking actionable plan to manage this communicable disease. Till now, ZIKV is considered an incurable infectious disease [2]. In the ongoing studies, researchers are aiming to develop a vaccine to prevent this infection. Some preclinical trials are studying an inactivated form of this virus, and the pharmaceutical industry promises that a product will soon be introduced to the market to combat the ill effects caused by this virus [10].

Because ZIKV is highly threatening and rapidly progressing, coupled with the fact that it can be transmitted through travelers between countries, it is highly necessary for healthcare providers (HCPs) to have sufficient knowledge about such a disease, infection control measures, and its management [11–13]. This is particularly important in the Kingdom of Saudi Arabia (KSA) as every year more than 2 million people visit the country from various regions of the world for pilgrimage, which increases the risk of infections with various communicable diseases including the ZIKV [14]. Hence, HCPs need to have sufficient knowledge about the widespread infectious diseases, their symptoms, modes of transmission, and methods of prevention and treatment. Although multiple studies have been published globally on assessing the knowledge regarding ZIKV among HCPs, this study is the first of its kind to be conducted in Saudi Arabia [9, 15–17]. The purpose of our study is to assess the knowledge of HCPs regarding ZIKV in Riyadh, KSA.

## 2. Methodology

### 2.1. Study Design and Sampling Technique

A cross-sectional survey study was conducted in Riyadh, KSA, during a two-month period from September 19, 2016, to November 19, 2016.

### 2.2. Sample Size Calculations

Assuming a desired precision level of 5% and a confidence interval of 95%, the current study required 353 healthcare professionals to be interviewed. Assuming a finite population of healthcare professionals of 2000 individuals, the finite population corrected sample size needed for the study was 278 subjects. The estimated sample size was also sufficient to determine the knowledge of ZIKV.

### 2.3. Study Setting and Subjects

This cross-sectional survey study was conducted in King Abdulaziz Medical City. It is a 1700-bed academic tertiary care center located in Riyadh, KSA. The survey was used to assess the knowledge of HCPs regarding ZIKV in Riyadh. We picked a sample in each department and manually distributed the survey to those who have been selected for the study. This study includes HCPs, who are currently working at the National Guard Health Affairs (NGHA) hospital, from different specialties (physicians, dentists, nurses, pharmacists, and nutritionists). In addition, the study focused on HCPs working in the following departments: medicine, surgery, dental, cardiac, pediatric, oncology, pharmacy, ambulatory care clinics, emergency room, and intensive care unit.

### 2.4. Questionnaire

This study is a cross-sectional study. It is based on a hard-copy questionnaire. The questionnaire was a slightly modified version of the one adapted from a previous study [9] and was assessed for validity and reliability in a sample of 20 HCPs; Cronbach's alpha measure of internal consistency was 0.78. It contains 23 questions, 5 demographical questions, and 18 multiple choice questions, with one correct answer, assessing the knowledge regarding Zika virus. Pilot participants have been included in the analyzed data. The administration process and gathering of filled questionnaires did not exceed a period of 1 week.

### 2.5. Ethical Considerations

Ethical approval (Ref. no. IRBC/838/16) was obtained from the IRB of the King Abdullah International Medical Research Center, NGHA, Riyadh, KSA. Participants consented to participate in the study before filling out the survey and could withdraw from the study at any time. The questionnaires were collected and securely stored in the principal investigator's office.

### 2.6. Statistical Analysis

Descriptive statistics were performed on data collected from the study sample. For continuous variables, data were expressed as means (standard deviation), medians, and ranges. Proportions were used to describe categorical variables. Knowledge scores based on 18 questions about ZIKV were evaluated and compared by demographic characteristics (age, designation, years of practice, and gender) using the *t*-test/Mann–Whitney *U* test or the Kruskal–Wallis test, as appropriate. The highest score achieved by any participant could be 18. The correct response was awarded a score of 1, while an incorrect response was given a score of 0. The knowledge score was calculated as the percentage of correctly answered knowledge questions (range 0–100), with higher scores indicating better knowledge.

## 3. Results

A total of 336 HCPs completed the questionnaire. Descriptive statistics of the respondents are displayed in Table 1. Most of the respondents were between 25 and 34 years of age, and 63% were females. The majority of respondents were from medicine department (35%) and nurse designation (48%). In addition, 290 (86%) respondents have less than 10 years of practice. The Internet was the most common source of knowledge regarding ZIKV (53%).

**Figure 1:**
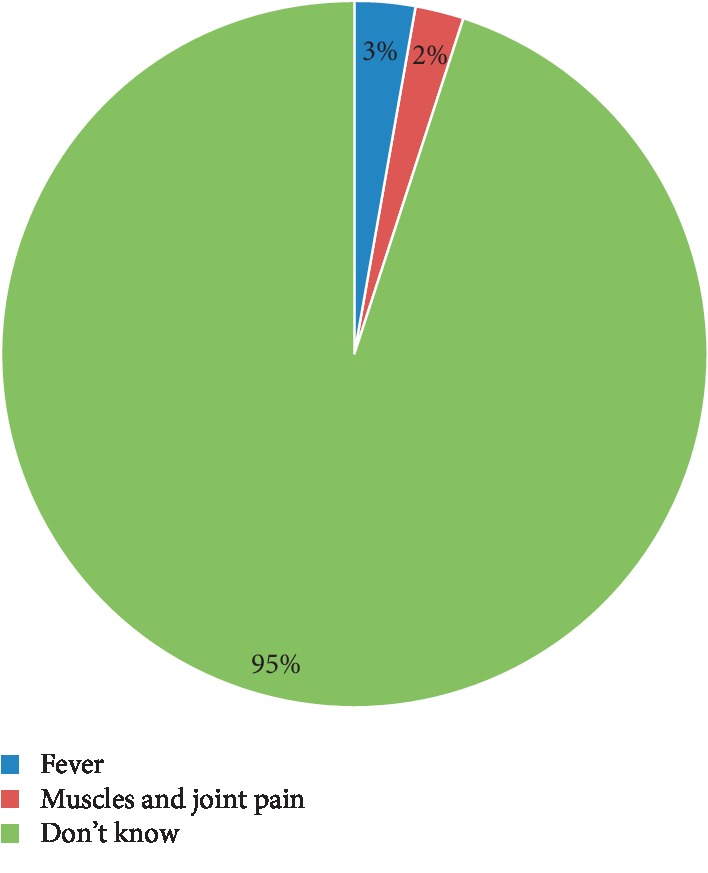
Respondents' knowledge regarding Zika virus signs and symptoms.

Table 2 shows the results from analyzing various responses of practitioners regarding ZIKV. It shows that 95% and 58% of practitioners believed that ZIKV is of viral origin and it is a communicable disease, respectively. Furthermore, 88% of respondents believed that it causes birth defects and 74% agreed that birth defects are mainly caused by microcephaly. Fever and muscle and joint pain signs and symptoms were reported only by 5% of respondents (Figure 1). Syndromes associated with ZIKV were reported by respondents as follows: Guillain-Barre syndrome (33%), Down's syndrome (20%), and Colic and crying syndrome (4%). More than half of HCPs reported that there are no available vaccines for ZIKV. Majority of respondents thought that ZIKV is transmitted by mosquitoes and not sexually transmitted.

Results of comparing knowledge scores by participants' demographic factors are shown in Table 3. Significant differences in knowledge about ZIKV were observed by participant's age and department. Significantly higher knowledge levels were observed among older participants (45 years or more;*p*=0.011). In addition, a substantial difference in the knowledge level was observed by department, with pairwise comparisons showing significant differences in knowledge scores between all departments except for Pharmacy vs. Nutrition and Nursing vs. IM.

Years of practice may slightly influence the level of knowledge. Practitioners who have 10 or more years of practice have slightly higher score compared with the other group, but it is not statistically significant.

## 4. Discussion

To the best of our knowledge, this study is the first of its kind to assess the knowledge regarding ZIKV among HCPs from different specialties (physicians, nurses, pharmacists, and nutritionists) in Saudi Arabia. In our study, there is a significant difference in knowledge between age groups and different departments. Participants who aged 45 years or more had more knowledge regarding ZIKV compared with younger participants. Compared with other specialties, internal medicine physicians were more knowledgeable about ZIKV which might be justified by the fact that this specialty is broader in nature than others and encompasses multiple areas. When it comes to years of practice, HCPs whose experience is 10 years or more had slightly more knowledge, but it was not statistically significant. Majority of the participants were aware that ZIKV is a communicable disease and of viral origin. Majority were also aware that pregnant women are the most vulnerable population to ZIKV and its ability to cause birth defects.

Recently, there are some studies conducted that addressed this issue [9, 15–19]. One of which was conducted among dentists in India [9]. In that study, a survey was distributed to 412 participants and showed that majority of participants (61.8%) had inadequate knowledge about ZIKV infection [9]. Furthermore, a study conducted among HCPs in Indonesia showed comparable results (50%) [16]. Another study conducted among Middle Eastern practitioners in Qatar found that among 446 participants, 66% reported poor knowledge regarding ZIKV [15]. Moreover, a study surveyed practitioners from different countries revealed that the majority of participants have an inadequate knowledge regarding ZIKV [17]. However, a recent study conducted among general practitioners (GPs) in Indonesia showed that 64% of them had good knowledge regarding ZIKV [18]. In addition, another recent study conducted among GPs in Indonesia showed that 66.5% of them had a good knowledge of pregnancy-related issues of ZIKV [19]. In our study, the majority of the respondents have an inadequate knowledge about ZIKV. Also, the knowledge level about ZIKV was significantly different by age; those aged 45 or more had significantly better knowledge than others. Lastly, a substantial difference in the knowledge level was observed by department, with pairwise comparisons showing significant differences in knowledge scores between all departments except for Pharmacy vs. Nutrition and Nursing vs. IM.

The inadequate knowledge of ZIKV infection among HCPs can be improved by intensifying the annual awareness raising Hajj campaigns that are conducted in the hospitals and enforced by the government which aim to face and prevent infections among pilgrims. Pilgrimage is an annual gathering of people in Mecca, KSA. Around two million pilgrims come every year from various countries around the world, which may carry a risk of emergence or transmission of infectious diseases [14]. Although ZIKV infection has not been reported in KSA, there are many pilgrims who annually visit from countries in which this infection was reported, so the risk of having this infection transmitted during pilgrimage cannot be ruled out.

Middle East respiratory syndromecoronavirus (MERS-coV) nosocomial outbreak was first reported in KSA in 2012 which derived huge governmental efforts on infection control and prevention in the past few years [20]. As a result, HCPs in KSA should be motivated to update their knowledge in the emerging infections as they just faced MERS-coV nosocomial outbreak in the country.

Regarding the source of knowledge of ZIKV, Internet was the most commonly used method (53%) among HCPs to obtain information about this infection. This result is consistent with previous studies conducted by Gupta et al. and Harapan et al. which revealed that Internet and online media were the main sources to obtain such information [9, 16]. This result is expected due to the availability of technology and smart phones which are frequently used in the medical field.

This study has some limitations: it was conducted in a single center in Riyadh, KSA, and most of respondents were from the medicine department and they were mainly physicians and nurses. However, our study is the first to address this issue in KSA and it included HCPs from different specialties and departments.

## 5. Conclusion

This study showed inadequate knowledge of HCPs from different specialties about ZIKV infection. Thus, knowledge about ZIKV should be improved in terms of route of transmission, clinical manifestations and diagnostic criteria, its potential complications, as well as infection prevention and control because ZIKV and other emerging infectious diseases are considered big threats to our country, particularly during the annual pilgrimage. Awareness about ZIKV infection should be ensured and maintained among HCPs to face any possible emergence in the region.

## Figures and Tables

**Table 1:** Demographic characteristics of the practitioners.

Factor	Number	Percent (%)
*Gender*		
Male	124	36.9
Female	212	63.1

*Age group (years)*		
25–34	185	55.06
35–44	80	23.81
45–54	32	9.52
55–64	21	6.25

*Department*		
Critical care/intensive care unit	16	4.8
Surgery	18	5.4
Emergency room	7	2.1
Medicine	118	35.1
Pediatrics	19	5.7
Oncology	13	3.9
Pharmacy	40	11.9
Ambulatory care clinics	9	2.7
Cardiac	64	19
Nephrology	32	9.5

*Designation*		
Physician	118	35.1
Nurse	162	48.2
Pharmacist	44	13.1
Nutritionist	12	3.6

*Years of practice*		
10 years or more	46	13.7
<10 years	290	86.3

*Sources of knowledge about Zika*		
Internet	177	52.7
Television	145	43.2
Others	14	4.2

**Table 2:** Responses of practitioners regarding Zika virus *N* = 336.

Item/question	Options	No. of responses	Percent (%)
ZIKV is of:	Bacterial origin	1	0.3
	Viral origin	320	95.2
	I don't know	15	4.5

ZIKV was first identified in:	1947	60	17.9
	1952	48	14.3
	2007	23	6.8
	2016	97	28.9
	I don't know	108	32.1

Sign and symptoms:	Fever	11	3.3
	Muscles and joint pain	6	1.8
	I don't know	319	94.9

Incubation period (days):	2–7 days	51	15.2
	7–14 days	122	36.3
	15–30 days	28	8.3
	I don't know	135	40.2

Does ZIKV cause birth defects?	Yes	296	88.1
	No	2	0.6
	I don't know	37	11.0

Birth defects caused by:	Aneurysm	3	0.9
	Microcephaly	248	73.8
	Cleft lip and cleft palate	4	1.2
	I don't know	81	24.1

Syndromes associated with ZIKV:	Zika is of Down's syndrome	68	20.2
	Guillain-Barre syndrome	112	33.3
	Colic and crying syndrome	14	4.2
	I don't know	142	42.3

Vector associated with ZIKV:	*Aedes aegypti*	167	49.7
	*Psorophora*	14	4.2
	*Mansonia titilans*	6	1.8
	*Anopheles*	36	10.7
	I don't know	113	33.6

ZIKV is primarily seen in:	Brazil (America)	161	47.9
	Columbia (America)	17	5.1
	Cape Verde (Africa)	77	22.9
	Samoa (Oceania/Pacific Island)	22	6.5
	I don't know	59	17.6

ZIKV got its name from:	Place of origin	89	26.5
	Founder of virus	44	13.1
	Species of mosquitoes	105	31.3
	I don't know	98	29.2

Is there any vaccine for ZIKV?	Yes	45	13.4
	No	194	57.7
	Maybe	31	9.2
	I don't know	66	19.6

ZIKV is a communicable disease?	Yes	194	57.7
	No	79	23.5
	Maybe	14	4.2
	I don't know	49	14.6

Is it a sexually transmitted disease?	Yes	93	27.7
	No	169	50.3
	Maybe	28	8.3
	I don't know	46	13.7

Know diagnostic test for ZIKV?	Yes	121	36.0
	No	90	26.8
	Maybe	25	7.4
	I don't know	100	29.8

Who are the most at risk for ZIKV	Women	5	1.5
	Children	17	5.1
	Pregnant women	257	76.5
	I don't know	57	17.0

The ZIKV transmitted by:	Mosquitoes	282	83.9
	Contaminated food and water	12	3.6
	I don't know	42	12.5

Is ZIKV a lethal?	Yes	184	54.8
	No	83	24.7
	I don't know	69	20.5

**Table 3:** Descriptive statistics and comparison of knowledge scores by participants' demographic factors.

	*N* ^*∗*^	Mean ± SD	Median (IQR)	*p* ^*∗*^
*Age (years)*				0.011
18–44	283	54.1 ± 21.1	58.8 (41.2–70.6)	
45 or more	53	62.0 ± 18.5	64.7 (52.9–76.5)	

*Gender*				0.85
Male	124	55.1 ± 21.8	58.8 (41.2–70.6)	
Female	212	55.5 ± 20.4	58.8 (47.1–70.6)	

*Department*				<0.001
Pharmacy	44	42.0 ± 22.2	41.2 (29.4–52.9)	
Nursing	162	59.5 ± 18.4	58.8 (52.9–70.6)	
IM	38	61.5 ± 20.0	64.7 (47.1–76.5)	
Non-IM	80	52.8 ± 21.6	58.8 (41.2–69.1)	
Nutrition	12	46.1 ± 23.4	55.9 (23.5–64.7)	

*Years of practice*				0.39
10 years or more	46	57.8 ± 19.4	61.8 (52.9–70.6)	
<10 years	290	55.0 ± 21.1	58.8 (41.2–70.6)	

^*∗*^Based on the *t*-test (for age, gender), Mann–Whitney *U* test (for years of practice), and Kruskal–Wallis test (for department). IQR, interquartile range; IM, Internal Medicine, Non-IM, Noninternal Medicine.

## Data Availability

The data used to support the findings of this study are available from the corresponding author upon request.
